# Substituting Fossil-Based into Bio-Based Isocyanates for Resin and Dispersion Polyurethane Coatings: Evaluation of Thermal, Mechanical, and Chemical Performance

**DOI:** 10.3390/polym17243301

**Published:** 2025-12-12

**Authors:** Pieter Samyn, Patrick Cosemans

**Affiliations:** Department of Innovations in Circular Economy and Renewable Materials, SIRRIS, Gaston Geenslaan 8, B-3001 Leuven, Belgium; patrick.cosemans@sirris.be

**Keywords:** polyurethane, coating, isocyanates, bio-based, polyester polyol, polyacrylate polyol, wear, degradation

## Abstract

This study investigates the substitution of fossil-based isocyanates with bio-based alternatives in polyurethane resin (PU) coatings and polyurethane dispersion (PUD) coatings, focusing on mechanical and thermal performance. The coatings were formulated using bio-based pentamethylene diisocyanate (PDI) and a range of fossil-based hexamethylene diisocyanate (HDI) trimers, combined with either a polyester polyol or a polyacrylate polyol. Differential-scanning calorimetry analysis revealed that PDI-based coatings exhibit higher reactivity during crosslinking, resulting in higher glass transition temperatures. Thermogravimetric analysis showed lower thermal stability compared to HDI-based polyurethanes, indicating increased rigidity but reduced thermal resilience. Mechanical testing of the coatings on wood showed superior microhardness, scratch resistance, and wear resistance for PDI-based coatings, particularly when combined with polyester polyols. Microscopic surface evaluation and roughness analysis confirmed smoother morphologies and lower crack densities in PDI-polyester coatings. Gloss and water contact angle measurements further demonstrated improved surface uniformity and hydrophobicity for PDI-based coatings. The FTIR spectroscopy validated the chemical integrity and more intense hydrogen bonding for PDI-based coatings. The post-wear spectra indicated chemical oxidation and surface rearrangements in PDI-based systems and mechanical degradation with chain scission for HDI-based coatings. Overall, the study highlights that bio-based PDI trimers can effectively replace fossil-based HDI trimers in PU and PUD coatings without compromising mechanical performance, especially when paired with polyester polyols. These findings support the development of more sustainable polyurethane coatings with enhanced durability and environmental compatibility.

## 1. Introduction

Polyurethane (PU) is widely applied in the industrial and consumer sectors as a coating and adhesive [1], due to its excellent mechanical durability, corrosion resistance, chemical resistance, and surface aesthetics [2]. PU coatings are traditionally synthesized from fossil-based isocyanates and polyols, which contribute to environmental concerns related to resource depletion and carbon emissions. In response to increasing sustainability demands, the development of alternative bio-based precursors for PU coatings has gained momentum [3,4]. In particular, the bio-based isocyanates derived from renewable feedstock are promising to partially replace fossil-based components in PU formulations. A range of isocyanates is economically viable from biomass-based sources for coating applications [5], but the non-phosgenic synthesis routes [6] and the development of efficient catalytic systems remain a challenge for the production of a green PU [7]. Alternatively, the difunctional isocyanates can be synthesized through phosgene-free routes [8], employing the reaction of amines with carbonates or urea derivatives [9]. In parallel, the use of solvents can be reduced by a switch towards waterborne polyurethane dispersion (PUD) coatings [10,11]. While life cycle environmental impact studies on PU and PUD coatings highly depend on the context [12], they demonstrated that the use of bio-based components can reduce their environmental impact. However, the additional development and performance optimization of bio-based crosslinkers were deemed crucial [13]. From an economic point of view, the higher cost of bio-based coatings should be balanced by finding operational conditions for enhanced performance and lifetime.

Recent advances in green chemistry have increased the availability of bio-based isocyanates. Pentamethylene-diisocyanate (PDI) can industrially be produced from starch sources (e.g., corn) that are broken down into sugars. The latter are used for the synthesis of the pentamethylene diamine intermediate in the presence of genetically engineered microorganisms. The PDI trimers can be effectively produced in a jet loop reactor [14] and purified by the appropriate double-effect distillation [15]. In parallel, the formation of high-molecular-weight oligomers that are formed through self-polymerization reactions of the diisocyanates can be hindered to reduce the viscosity of PDI trimers [16]. The PU films produced from bio-based isocyanates, such as l-lysine ethyl ester diisocyanate (LDI), contribute to a higher flexibility and provide a more ductile material as compared to the fossil-based isophorone diisocyanate (IPDI) [17]. As compared to the LDI and hexamethylene-diisocyanate (HDI), the PDI trimer resulted in more reactive PU formulations with the highest crosslinking density and thermal stability [18]. The PDI trimer curing agent has lower-life-cycle carbon emissions than the HDI trimer curing agent, and generally provides coatings with good water resistance, chemical resistance, and aging stability [19]. The formulations of PUD coatings using PDI as the hard segments confirmed that increasing the molar ratio with the polyether polyol could improve the extent of hydrogen bonding and glass transition temperature [20]. However, the selection of diisocyanates plays a fundamental role in the morphology of films and coatings made from waterborne polyurethane dispersion (PUD) [21], where interactions between the colloidal particles determine coating homogeneity and mechanical properties [22].

The present study provides a comprehensive evaluation of polyurethane resin coatings (PU) and polyurethane dispersion coatings (PUD), formulated with bio-based pentamethylene diisocyanate (PDI) trimers and fossil-based hexamethylene diisocyanate (HDI) trimers. Unlike previous works that focus on isolated components or single coating types, the novelty of the present study lies in a broader approach that is rarely addressed in such depth, enabling a more holistic understanding of structure–property relationships of both resin and dispersion coatings. The study integrates the effects of isocyanate substitution (PDI vs. HDI) and polyol chemistry (polyester, polyacrylate polyols) to assess thermal, mechanical, and surface properties. By incorporating polyester versus polyacrylate polyols within resin or dispersion coatings, the study reveals how polyol chemistry interacts with isocyanate types to influence glass transition temperatures, hardness, wear resistance, and hydrophobicity. New insights into degradation mechanisms and surface rearrangements are revealed from postmortem FTIR and hydrophobicity analysis. The findings ultimately demonstrate that bio-based PDI trimers can enhance coating performance while supporting sustainable material development, marking a significant advancement in evaluating bio-based alternatives for high-performance polyurethane coatings.

## 2. Materials and Methods

### 2.1. Materials

**Figure 1:**
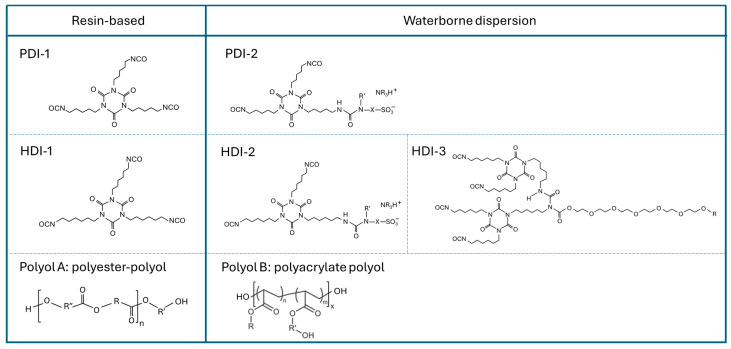
Components of isocyanates (PDI-1, PDI-2, bio-based; HDI-1, HDI-2, HDI-3, fossil-based) and polyols (polyester polyol A, polyacrylate polyol B) for formulation of polyurethane coatings.

The different monomers for polyurethane coatings were selected according to Figure 1, including the following types of isocyanates: PDI-1 (Desmodur eco N7300), PDI-2 (Bayhydur eco), HDI-1 (Desmodur ultra), HDI-2 (Bayhydur XP), HDI-3 (Bayhydur ultra); and two types of compatible polyols: polyester polyol A (Desmophen 1300 BA), polyacrylate polyol B (Bayhydrol A2651). All materials were kindly supplied by Covestro (Leverkusen, Germany). The 1,5-pentamethylene-diisocyanate (PDI) trimer contains 68 ± 4% renewable carbon; the 1,6-hexamethylene diisocyanate (HDI) trimer is a 100% fossil-based product.

### 2.2. Coating Formulation

The coating formulations were prepared according to the compositions in Table 1, including the PU coatings (PDI-1, HDI-1, and polyol A) and PUD coatings (PDI-2, HDI-2, HDI-3, and polyol B). The bio-based isocyanates were combined in different weight ratios with the fossil-based isocyanates, while mixing with a stoichiometric ratio of the polyester polyol A or polyacrylate polyol B (NCO/OH = 1/1). The effective weight of both components was calculated from the isocyanate equivalent weights (IEW) of the isocyanate mixtures and hydrogen equivalent weight (HEW) of the polyols (manufacturer’s data), as follows: PDI-1 (% NCO = 22, IEW = 195 g/eq), PDI-2 (% NCO = 18, IEW = 232 g/eq), HDI-1 (% NCO = 22, IEW = 195 g/eq), HDI-2 (% NCO = 20, IEW = 205), HDI-3 (% NCO = 16.2, IEW = 260), polyol A (% OH = 2.3, HEW = 530 g/eq), polyol B (% OH = 3, HEW = 566 g/eq).

The coating formulations were prepared in small batches by first mixing the isocyanate combination PDI + HDI for 3 min, followed by adding the polyol and gently mixing for another 5 min. The coating was placed into a vacuum chamber for deaeration. The coatings were applied onto the hardwood (beech wood) substrates by blade coating at a speed of 5 mm/s. The wet coating thickness was controlled by a metering bar of 500 µm, corresponding to a dry coating thickness of 450 ± 5 µm. The coatings were cured under controlled ambient conditions (50% RH, 23 °C) for 20 days before testing.

### 2.3. Characterization Methods

The curing characteristics and thermal properties of the coatings were analyzed by differential scanning calorimetry (DSC) on a DSC 3+ equipment (Mettler Toledo, Zaventem, Belgium). During the first heating, the liquid samples (4 mg) were loaded in a hermetic aluminum pan and sealed while heating from 20 °C to 120 °C at a rate of 20 °C/min under a nitrogen atmosphere. The crosslinked samples were subjected to a second heating cycle between −100 and 80 °C at 20 °C/min, also under nitrogen flow. Thermogravimetric analysis (TGA) was carried out on a TGA-1 equipment (Mettler Toledo, Columbus, OH, USA), where samples of 5 mg were heated up to 600 °C at a rate of 20 °C/min under a nitrogen flow of 60 mL/min.

The mechanical coating performance was evaluated by standardized testing methods known to the coating industry. The abrasive wear testing was performed on a rotating Taber tester Model 5130 (Taber Industries, New York, NY, USA) while loaded under 250 g or 500 g against the calibrated CS-10 abrasive wheels, following ASTM D4060-10 [23]. The weight loss was measured after a total of 1000 cycles on an analytical balance with 0.001 g precision (Sartorius, Göttingen, Germany). The wear testing data was averaged over three samples (*n* = 3) and reported as the average value with error bars corresponding to the standard deviation. The indentation microhardness was tested with a handheld Shore D micro-indenter containing a hardened steel tip (30° angle, 0.1 mm radius), following ASTM D2240-15 [24]. The hardness values were measured over ten locations of the coating (*n* = 10), and reported as an average value with error bars corresponding to the standard deviation. The scratching tests were performed with a sclerometer Type 3092 with a tungsten carbide tip of 0.75 mm radius (Elcometer, Aalen, Germany), inserting a spring under 10 or 20 N normal load according to ISO 4586-2 [25]. The surface gloss was measured with a micro-glossmeter (BYK-Gardener Instruments, Geretsried, Germany) under a 60° incident light angle according to ISO 2813 [26]. The gloss values were measured over ten locations of the coating (*n* = 10), and reported as an average value with error bars corresponding to the standard deviation. Visual inspection of the surfaces was performed with an optical digital microscope VHX-7000 (Keyence, Mechelen, Belgium) at low magnification (5×), while a more detailed topography of worn surfaces was observed with a confocal laser interference microscope VK-X3000 (Keyence, Mechelen, Belgium) at higher magnification (20×, 50×, 150×). Static water contact angles were measured with an OCA 50 system (Dataphysics Instruments GmbH, Filderstadt, Germany), following ISO 19403-2 [27]. The static water contact angles were determined from the deposition of 3 µL droplets with geometries analyzed via tangent fitting. The contact angles were measured over ten locations of the coating (*n* = 10), and reported as an average value with error bars corresponding to the standard deviation.

The spectroscopic evaluation of initial coatings and worn coatings was performed by attenuated total reflection Fourier transform infrared spectroscopy (ATR-FTIR) on a Nicolet iS10 spectrometer with a HeNe laser and a diamond crystal (Thermo Fischer, Breda, The Netherlands). The spectra were collected at 600 to 4000 cm^−1^ wavenumbers with a 4 cm^−1^ resolution and averaged over 16 scans.

## 3. Results

### 3.1. Thermal Evaluation

**Figure 2:**
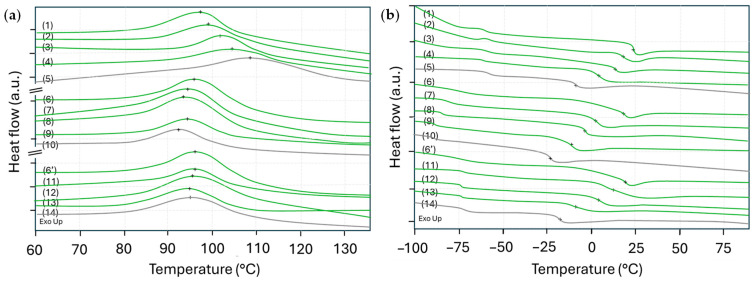
DSC curves of polyurethane coatings, (**a**) first heating of liquid samples showing exothermal reaction during curing with temperature of maximum intensity (+), (**b**) second heating of the crosslinked samples showing glass transition Tg (+). Sample numbers refer to Table 1, including bio-based grades (green) and fossil-based grades (grey).

First, the curing properties of the polyurethane coatings were evaluated by DSC measurements on the liquid samples, monitoring the exothermal reaction during crosslinking between the isocyanates and polyol (Figure 2a). The progressive shifts in temperatures corresponding to the maximum intensity of the exothermal peak indicate influences of the bio-based PDI versus fossil-based HDI monomers. For the PU coatings, the maximum exothermal peak of PDI formulations occurs at lower temperatures compared to the HDI formulations. This indicates the higher reactivity of PDI-1 monomers as compared to the HDI-1 monomers, depending on the higher availability of reactive isocyanate groups per weight fraction. For the PUD coatings, the crosslinking is also observed with smaller variations between PDI and HDI monomers. At least the intensity of the exothermal peak is higher for crosslinking of coatings with PID-2 monomers as compared to HDI-2 and HDI-3 monomers, while it might happen at a slightly lower temperature. The crosslinking of coatings with HDI-3 monomers at slightly lower temperatures than coatings with HDI-3 monomers may indeed be related to the higher accessibility of the reactive isocyanate groups linked to flexible aliphatic chains in HDI-3. Overall, the more difficult crosslinking of coatings deposited from aqueous dispersion may be expected due to effects such as, e.g., diffusion of the reactive moieties and availability of reactive groups. In particular, polymer diffusion between dispersion particles is a very slow process, resulting in limited availability and mobility of reactive groups, which hinders effective crosslinking in aqueous systems [28]. The curing conditions for PUD coatings with amino functional groups allow for good film-forming properties at room temperature [29].

During the second heating of the crosslinked samples, the glass transition temperatures Tg (Figure 2b) related to the soft segment (polyol segment) and hard segment (isocyanate segment) of the polyurethane molecules are clearly observed. The Tg related to the soft segments occurs at temperatures of −58 to −63 °C for PU coatings (samples 1 to 5), −68 to −73 °C for PUD coatings (samples 6 to 10), and −75 to −78 °C for PUD coatings (samples 6′ to 14). As related to the soft segments, the Tg values in the low-temperature region depend on the molecular structure of the polyol [30]. The polyester polyol A is characterized by a more rigid backbone due to ester linkages and a linear structure, introducing stronger intermolecular interactions like hydrogen bonding. The polyacrylate polyol B typically has more branched side chains with less cohesive energy densities, introducing higher flexibility and rotational molecular freedom. Therefore, the high rigidity of the polyester polyol A segments corresponds to a higher Tg as compared to the polyacrylate polyol B segments. This follows the general influences of the chemical structure of polyols on Tg [31]. Typically, the transitions related to the soft segments are relatively weak and more sensitive to observe. There is a consistent shift in Tg values of the hard segments varying between the compositions with PDI and HDI monomers. Overall, the polyurethane coatings with bio-based PDI monomers have a higher Tg as compared to the fossil-based HDI monomers. The progressive decrease in Tg with higher concentration of HDI monomer indicates that there is good compatibility and homogeneous mixing between the different isocyanate types. It can indeed be expected that the more compact structure of the aliphatic chains in the PDI-1 or PDI-2 trimer (5 carbons) introduces higher rigidity, while the longer side chains in the HDI-1 or HDI-2 trimer (6 carbons) favor the molecular mobility. For PUD coatings with polyacrylate polyol B, the higher Tg for coatings with HDI-3 trimer as compared to the HDI-2 trimer reflects the favorable effects of the molecular structure of the HDI-3 trimer; as indicated by the higher Tg, the higher concentration and availability of isocyanate groups in the HDI-3 trimer may increase the crosslinking density and formation of a more rigid polymer network. In conclusion, the same trends with the higher Tg of bio-based PDI monomers as compared to fossil-based HDI monomers are observed for PU and PUD coatings with either polyester polyol or polyacrylate polyol.

**Figure 3:**
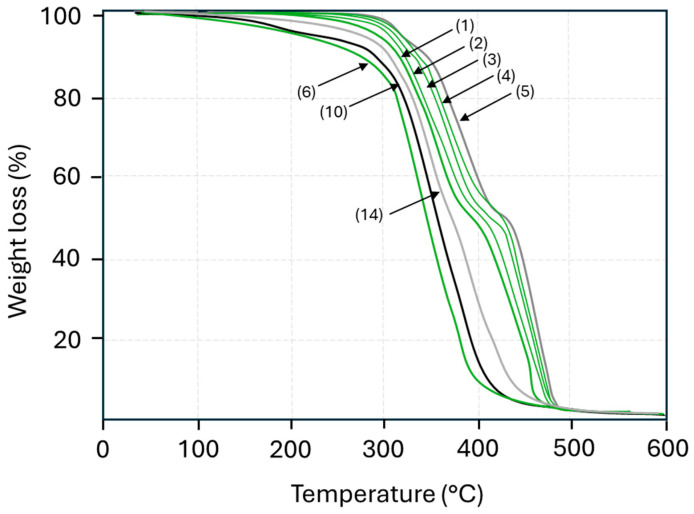
TGA curves of polyurethane coatings, including a representative set of samples. Sample numbers refer to Table 1, including bio-based grades (green) and fossil-based grades (black, grey).

The thermal stability of the PU and PUD coatings, as determined by the TGA measurements (Figure 3), indicates a progressive shift in degradation temperatures depending on the isocyanate type (PDI versus HDI) and polyol type (resin-based coatings with polyester polyol A, versus dispersion-based coatings with polyacrylate polyol B). The thermal decomposition of polyurethanes typically occurs as a multi-step reaction corresponding to the partial degradation of the hard segments (urethane linkages) at temperatures between 250 and 350 °C and the degradation of the soft segments (polyol linkages) at temperatures of 350 to 450 °C [32]. The PUD coatings with polyacrylate polyol B tend to produce coatings with lower thermal stability as compared to the PU coatings with polyester polyol A, as the degradation may start at around 200 to 250 °C (coatings with polyacrylate polyol B), with major degradation between 350 and 390 °C (coatings with polyacrylate polyol B), or 420 to 470 °C (coatings with polyester polyol A).

The first degradation step typically involves the cleavage of the urethane linkage (–NH–CO–O–), releasing isocyanates and alcohols [33]. For the PU coatings, the degradation of coatings with PDI-1 trimer occurs at a lower temperature compared to the coatings with HDI-1 trimer. The PU with HDI trimer shows better thermal stability, likely due to the longer aliphatic chain [34]. As the PU with PDI trimer degrades earlier, it may be advantageous for applications requiring controlled thermal breakdown or recyclability. For the same reasons in PUD coatings, the PDI-2 trimer gives lower thermal stability as compared to the HDI-2 trimer and HDI-3 trimer. The effects of the isocyanate structure on thermal stability are clearly expressed with a higher stability of HDI-3 as compared to the HDI-2 trimer. Indeed, the more flexible structure of the HDI-3 imparts less internal molecular stresses that better stabilize the molecular structure.

The second degradation step related to the polyol segments typically involves chain scission, producing volatile products like CO, CO_2_, and hydrocarbons. The PU coatings with polyester polyols may show strong intermolecular interactions (hydrogen bonding) and form more rigid backbones due to ester linkages that contribute to higher thermal stability. The multifunctional hydroxyl groups in the polyester polyol increase intermolecular forces due to the polar ester groups. Therefore, the PU coatings with polyester polyol segments are thermally more stable as the ester bonds resist thermal cleavage. In comparison, the polyacrylate polyols have more flexible chains and side chains with less cohesive energy density that are accessible for degradation and chain scission. The carbon–carbon bonds in the backbone of acrylates degrade more easily than the ester backbone of polyesters. Moreover, the lower polarity and bulkiness of acrylate groups lead to lower intermolecular forces. However, it is sometimes observed that acrylate chains possess higher thermal stability as compared to polyester chains [35]. Although polyester polyols degrade primarily via hydrolysis of ester bonds, they are better stabilized through hydrogen bonding as compared to the acrylate polyols [36]. The comparative thermal stability between PUD coatings with polyester polyol and polyacrylate polyol is in line with the results from DSC measurements.

### 3.2. Mechanical Evaluation

**Figure 4:**
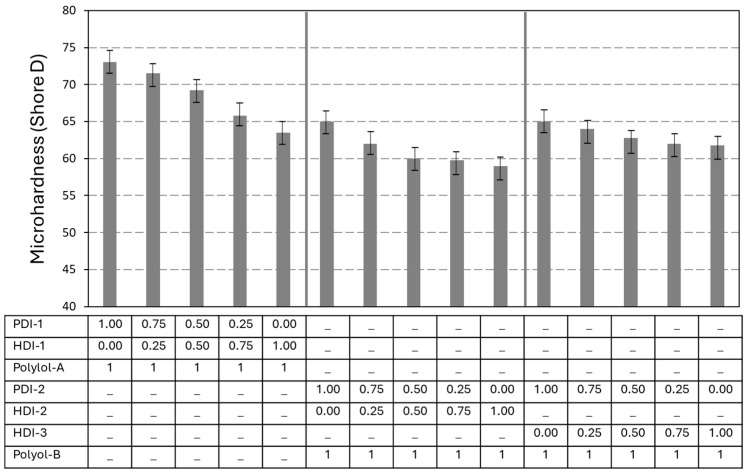
Microhardness testing results of polyurethane coatings (*n* = 10, error bars representing standard deviation).

The microhardness of the PU and PUD coatings was determined as a reference measurement for mechanical resistance (Figure 4). For all coatings, the microhardness of coatings with bio-based PDI trimers is superior as compared to that of coatings with fossil-based HDI trimers, with a progressively linear decrease in microhardness for the mixed isocyanates. The microhardness values for PU coatings with PDI versus HDI trimers are in line with the Tg values and related to the effects of molecular mobility as explained before. The better mechanical properties of the PU coatings with bio-based PDI against HDI are confirmed in parallel with the higher reactivity of PDI trimers.

The PU coatings formulated with polyester polyol A and polyacrylate polyol B exhibit distinct microhardness characteristics due to differences in their chemical structure, crosslinking density, and segmental mobility. The PU coatings with polyester polyol A generally have a higher microhardness than coatings with polyacrylate polyol B due to better crystallinity and hydrogen bonding, as also reflected in the higher Tg as determined from DSC. The PU coatings with polyester polyol A generally show higher crosslinking density, greater hardness, and higher Tg, which also corresponds to the greater mechanical and microbial resistance [37]. In contrast, the PUD coatings with polyacrylate polyol B generally have a lower microhardness in parallel with the lower Tg as determined from DSC. In agreement with earlier structure–property relationship studies on waterborne PUD films comparing polyacrylate emulsions and polyester dispersions, polyacrylate systems have a higher equivalent weight than polyester polyol, contributing to lower crosslinking density and softer films [38,39]. The higher molecular weight and amorphous nature of PUD films may reduce segmental packing, leading to softer mechanical properties, as studied previously [40]. Consequently, the crosslinking density of the polyacrylate-based PUD systems is often lower compared to that of the polyester-based PU systems, as observed in previous studies on polyurethane films: the polyacrylate-based films that had lower Tg were softer due to their amorphous nature and higher molecular weight [41]. Their hardness and Tg highly depend on the acrylate content and molecular weight, and are generally lower than polyester-based PU systems [42,43].

**Figure 5:**
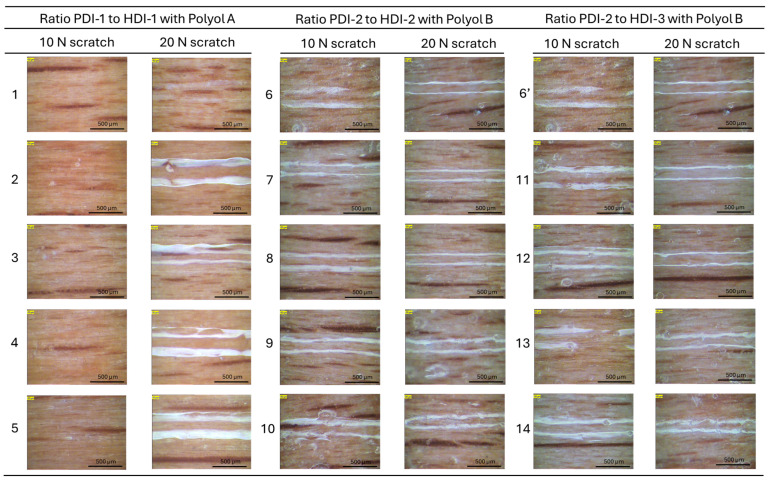
Scratching tracks under 10 N and 20 N on polyurethane coatings, visualized by optical microscopy (scales added in figures, bottom-right). Sample numbers refer to Table 1.

The results for scratch resistance testing on coatings under 10 N and 20 N (Figure 5) show the behavior as a function of bio-based PDI content and polyol types. For both PU coatings with polyester polyol A and PUD coatings with polyacrylate polyol B, the scratching damage on PDI-based coatings is less compared to the HDI-based coatings, which is in agreement with the previous results of a higher microhardness for PDI-based coatings. Overall, the PU coatings with polyester polyol A have superior scratch resistance compared to the PUD coatings with polyacrylate polyol B, which is in line with both the higher hardness and higher Tg values for PU coatings with polyester polyol A. The observations are confirmed by previous studies [44]; the scratch resistance of waterborne PU dispersion clearcoats was dependent on the base resin Tg, and the coatings with higher Tg and optimized crosslinking showed superior scratch resistance. Another study evaluated the scratch resistance of aqueous two-component polyurethane coatings [45]; it showed that scratch resistance varied with applied load and substrate compliance, and was influenced by the mechanical properties linked to Tg. The scratch resistance of the polyurethane coatings could indeed be tuned by adjusting the composition and Tg of the isocyanate [46].

**Figure 6:**
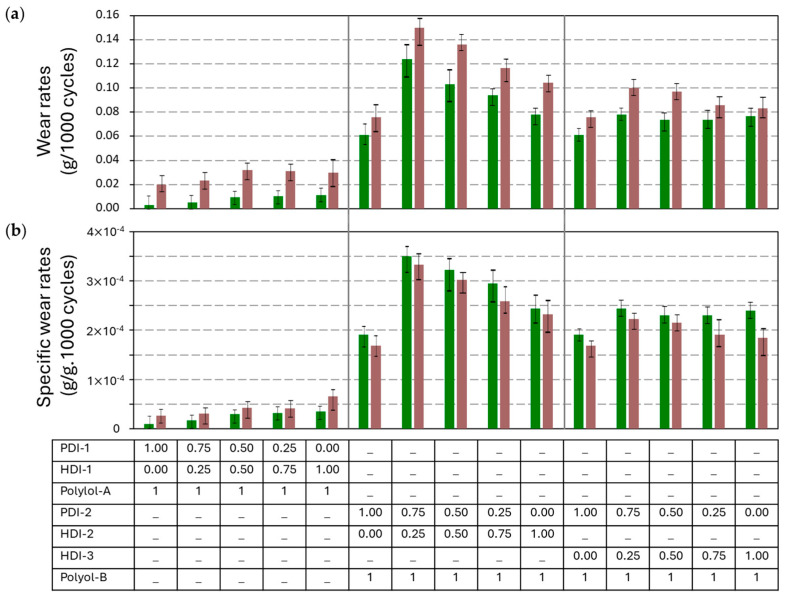
Abrasive wear testing results of polyurethane coatings under low load (250 g, green bars) and high loads (500 g, brown bar), (**a**) weight loss, (**b**) specific wear rates (*n* = 3, error bars representing standard deviation).

The weight loss of the coatings after abrasive wear was converted into wear rates (Figure 6a) and specific wear rates (Figure 6b), after testing under low loads of 250 g and high loads of 500 g. As expected, the specific wear rates under both low and high loads are in good agreement, as similar wear mechanisms within mechanical degradation under both loads may apply [47]. In parallel with the hardness measurements before, the wear rates of PU coatings with bio-based PDI-1 trimers are lower compared to the PU coatings with fossil-based HDI-1 trimer. The wear rates of the PUD coatings with HDI-2 trimers are higher than for the coatings with the HDI-3 trimers, in parallel with previous hardness and Tg measurements.

The wear rates for PU coatings with polyester polyol A are evidently lower as compared to the wear rates for PUD coatings with polyacrylate polyol B, in agreement with the hardness and Tg measurements. In particular, the studies before also revealed that polyester-polyol-based PU coatings showed enhanced wear resistance due to hydrogen bonding and higher cohesive strength, while polyacrylate-based PUD coatings tend to exhibit higher wear rates as they are more amorphous and flexible [48]. However, the new insights from this study for polyacrylate-polyol PUD coatings indicate that a lower hardness may result in better abrasive wear resistance. As the harder materials tend to be more brittle, they can lead to microcracking or fragmentation under abrasive stress. The softer PUD coatings with HDI monomers can likely deform plastically under abrasive contact while absorbing energy and reducing material loss. This energy dissipation mechanism helps prevent the propagation of wear tracks or gouges, especially under low to moderate load conditions. This phenomenon is often observed in polyurethane elastomers, where lower hardness grades show better wear resistance due to their ability to conform to abrasive particles rather than resist them rigidly [49].

**Figure 7:**
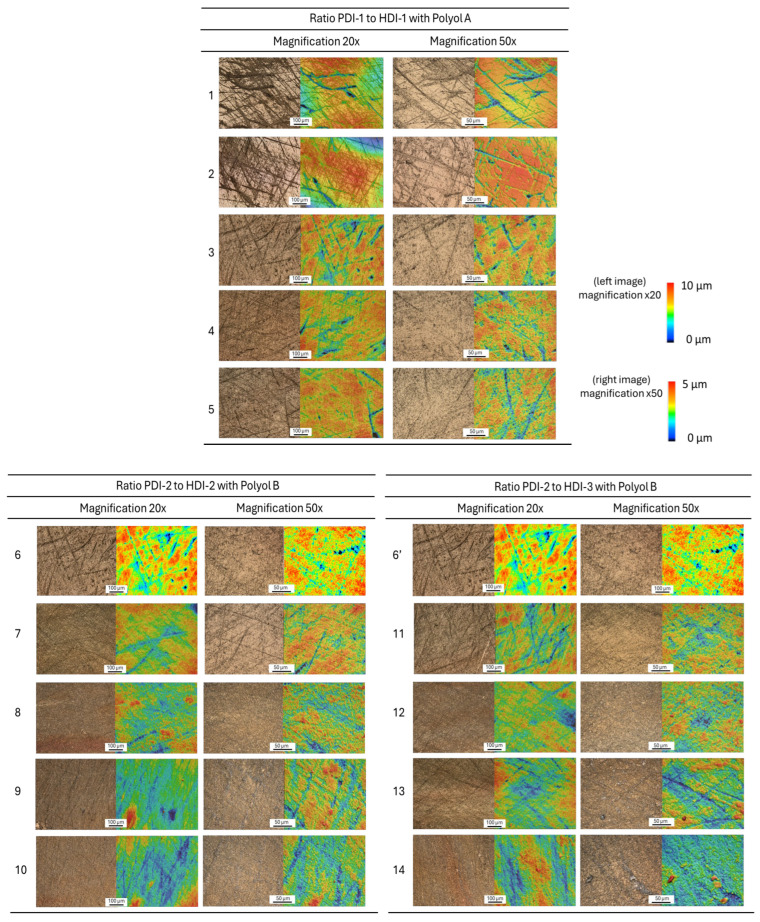
Microscopic evaluation of the wear tracks on polyurethane coatings, including optical micrographs and 3D surface topography under two different magnifications (same color scale for z-height applies, as indicated). Sample numbers refer to Table 1.

**Figure 8:**
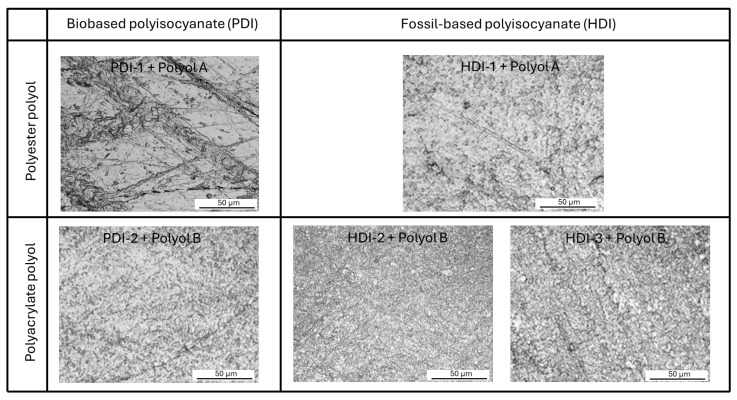
Detailed optical microscopy of some worn polyurethane coatings.

Based on the images of the worn coating samples (Figure 7), the morphology of the worn surfaces varies significantly depending on the PDI/HDI ratio and the polyol type. For the coatings with different ratios of PDI-1 to HDI-1 and polyester polyol A, the abrasive scratches and micro-cracks are visible, only attacking the superficial coating layer without full removal of the entire top coating layer. The local penetration of the abrasives and groove formation indicates a relatively brittle response under mechanical stress, in relation with the high surface microhardness or higher Tg values indicating a higher crosslink density. For the coatings with different ratios of PDI-2 to HDI-2 and polyacrylate polyol B, the wear tracks are smoother. There is less tendency for cracking and more plastic deformation, while some areas show material smearing in parallel with a more ductile behavior. These observations suggest improved toughness and elasticity, likely due to a more flexible polyol backbone or optimized isocyanate ratio. The results are in line with the reduced surface microhardness and lower Tg values. Similarly, for the coatings with a different ratio of PDI-2 to HDI-3 with polyacrylate polyol B, the pronounced plowing marks and material removal indicate material flow and scratch recovery in some regions. The latter observations indicate a coating behavior with higher viscoelasticity and energy dissipation, possibly due to lower hardness in line with lower Tg values. In conclusion, the coatings with higher Tg and crosslink density tend to produce harder, more brittle surfaces with crack-prone wear. The coatings with lower Tg or more flexible segments promote ductile wear with smoother morphology and better scratch recovery. The polyol type significantly influences the microstructure and wear behavior, affecting the coating’s ability to resist mechanical damage. These different surface features are further detailed by observations of the wear tracks at a higher magnification (Figure 8).

A quantitative analysis of the surface roughness and crack density is summarized in Table 2.

The average surface roughness Sa is determined from the topographical images at 20× magnification after standard surface flattening procedures. The algorithm used for crack density determination is based on image processing, including edge detection of grayscale images that are processed according to the Canny edge algorithm, which identifies sharp intensity changes typically corresponding to cracks or boundaries on the surface. A low- and high-threshold value is used to filter out noise and retain significant edges, while a binary image where white pixels represent detected edges is produced. The binary edge image is analyzed using connected component analysis, which groups adjacent edge pixels into distinct regions. Each region is assumed to represent a crack or crack-like feature, and the number of labeled regions is counted to estimate crack density. The crack density measured by the number of connected regions provides a quantitative estimate of how fragmented or damaged a surface is. A higher number means that the surface has more individual cracks as an indication of greater mechanical degradation. As a result, the PDI-1/HDI-1 + polyester polyol A coatings present a balanced profile with the lowest roughness and low crack density. The PDI-2/HDI-2 + polyacrylate polyol B coatings show the highest surface roughness and highest crack density in parallel with the more pronounced texture or microstructural variation, indicating more fragmentation or stress concentration. The PDI-2/HDI-3 + polyacrylate polyol B coatings have intermediate crack density and roughness. The quantitative analysis indeed aligns with previous, more qualitative descriptions of surface morphologies in relation to hardness and Tg of the respective coatings.

### 3.3. Optical Evaluation

**Figure 9:**
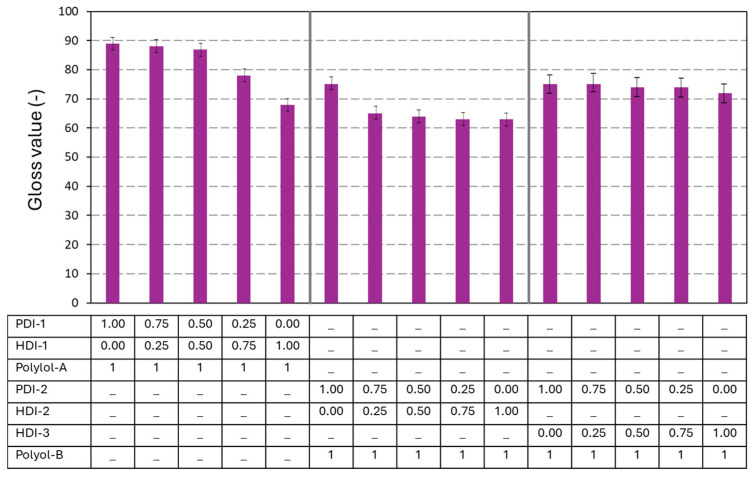
Gloss measurements on polyurethane coatings (*n* = 10, error bars representing standard deviation).

The gloss properties of the PU and PUD coatings were measured before wear (Figure 9), reflecting the surface morphology and uniformity in film formation. For the PU and PUD coatings with bio-based PDI trimer, relative to the PU and PUD coatings with fossil-based HDI trimer, typically higher gloss is measured for the PDI coatings. The latter tend to form more tightly crosslinked networks, resulting in smoother and more uniform surfaces that reflect light more effectively. This is in agreement with the higher reactivity of the PDI monomers observed in DSC measurements. For the HDI-based coatings with lower Tg, the polymer chains remain more flexible and rubbery at room temperature. This can cause surface deformation, micro-roughness, or matte finishes [50]. The systems with higher Tg often have higher crosslinking density, which stabilizes the surface and prevents phase separation. The phase-separated domains in low Tg systems could scatter light and reduce gloss [51].

Generally, the PU coatings with polyester polyol A present higher gloss due to the smooth and rigid surface with better flow and leveling. Polyester-based systems have lower segmental mobility and form more tightly crosslinked networks, which helps maintain a flat and reflective surface. The PUD coatings with polyacrylate polyol have lower gloss due to the slightly softer surface with a more matte or semi-gloss finish. The polyacrylate systems, with higher molecular weight and flexibility, may allow for slight surface deformation, scattering light, and reducing gloss. The higher surface tension of polyacrylate polyols may also lead to the formation of heterogeneities and a less uniform surface [52].

### 3.4. Hydrophobic Evaluation

**Figure 10:**
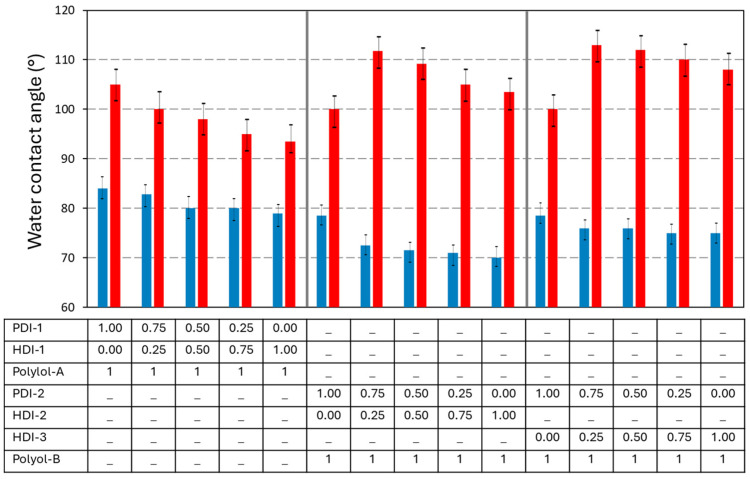
Static water contact angles on polyurethane coatings in native state before wear (blue bars), and in the wear track after abrasive wear (red bars), (*n* = 10, error bars representing standard deviation).

The water contact angles on the PU and PUD coatings before and after wear (Figure 10) confirm the effects of bio-based isocyanate content on hydrophobic protection.

The native PU coatings do not present hydrophobic properties, with water contact angles below 85°, depending on both the nature of the isocyanate and polyol parts. The PUs made with polyester polyols generally have better water resistance than those made with polyacrylate polyols [53], as also experienced in our tests. Although the ester linkages in the polyols are polar and can attract water molecules, the specific chemical makeup can alter this behavior: the presence of esterified hydrocarbon polymer chains as derived from fatty acids, in particular, makes the polyols more hydrophobic. The more compact structure of the polyester polyols with intrinsic hydrogen-bonding interactions, as confirmed in the previous mechanical testing, provides reduced access to the hydroxyl groups in their molecular structure. Otherwise, the polyacrylate polyols have a more polar character and contain fewer long, non-polar chains. The acrylic polymer backbone typically contains shorter segments that are less dominated by hydrophobic segments. Moreover, the PU coatings with bio-based PDI-trimers have a higher hydrophobicity as compared to the ones with fossil-based HDI-trimers, in line with the shorter aliphatic (non-polar) chains and higher Tg for the former formulations. The more ordered molecular arrangement for coatings with bio-based PDI-trimers may enhance the hydrogen bonding between the hard segments and therefore reduce the accessibility of hydroxyl groups for water interactions. The tight packing and extensive hydrogen bonding in PDI-based PU coatings reduce pathways for water penetration within the coating over time. Indeed, the more ordered molecular arrangements generally enhance hydrogen bonding between hard segments and reduce hydroxyl accessibility for water interactions, as the hydroxyl groups are involved in intra-/intermolecular hydrogen bonding within crystalline domains [54].

The water contact angles on the worn coatings have increased as compared to the native coatings, showing enhanced hydrophobic protection of the coatings after wear, likely owing to the combination of altered exposure of chemical moieties and change in surface topography after wear. In general, the hard segments from isocyanates are more polar and may be preferentially oriented towards the surface in order to enhance mechanical hardness and durability [55], while the soft segments (from polyols) are generally less polar [56]. Based on Hansen Solubility Parameters (HSPs), it was previously demonstrated that polyester polyols exhibit relatively low polarity compared to other components in polyurethane systems, including polyacrylate polyols [57]. If the more polar hard groups are preferentially removed through abrasion, the exposure of the non-polar soft segments may partially improve hydrophobicity. Therefore, different trends in contact angles on worn coatings are observed mainly depending on the used polyol, with polyacrylate polyols presenting the higher hydrophobicity on worn coatings in contrast to the polyester polyols [57]. For the worn PU coatings with polyester polyol A, the original trends with the higher hydrophobicity for coatings with PDI-trimers as compared to HDI-trimers remain. For the worn PUD coatings with polyacrylate polyol B, the hydrophobic properties of polyacrylate polyols prevail over the selection of the isocyanate monomers.

### 3.5. FTIR Spectroscopy

**Figure 11:**
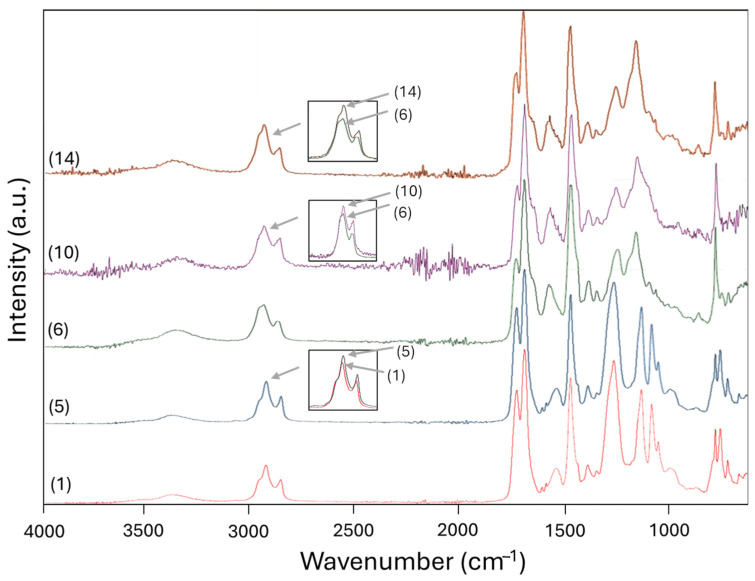
FTIR spectra of selected polyurethane coatings. Sample numbers referring to Table 1 (insets show detailed band overlaps for the aforementioned sample numbers in the region 2937–2850 cm^−1^).

A selection of FTIR spectra of native PU and PUD coatings is presented in Figure 11, and a full overview of the spectra for all samples is included in Supplementary Information S1. The presence of characteristic spectral bands of polyurethane can be confirmed in agreement with the literature [58], as further interpreted below.

The bands related to the isocyanate (–N=C=O) at 2260 cm^−1^ and polyol (–OH) at 3600 cm^−1^ were not observed [59], instead the crosslinking of the polyurethane coatings is confirmed by the characteristic bands that include the urethane groups in the range of 3400–3300 cm^−1^ [60], and the amide I band in the range of 1740–1680 cm^−1^ corresponding to the C=O vibration [61]. The band intensity might be slightly higher and shifted to the lower wavenumbers for the PU coatings with polyester polyol, owing to the enhanced interaction by hydrogen bonding.

The region at 1530–1540 cm^−1^ is typically assigned to two overlapping vibrations of the urethane CN–H group (amide II), including the in-plane bending vibration for N–H (1510–1570 cm^−1^) and a stretching vibration for C–N (1530–1540 cm^−1^) [62]. The N–H bending is often stronger than the C–N stretching, especially in hydrogen-bonded environments. As the respective bands for the PU coatings with polyester polyols A are shifted to the lower wavenumbers, this corresponds to the stronger hydrogen bonding and crosslinking densities. The polyester polyols typically have linear or branched aliphatic chains with ester groups, which promote interchain hydrogen bonding. The upward shift in bands in this region for the PUD coatings with polyacrylate polyols B confirms that there is a reduced hydrogen bonding due to steric hindrance or lower polarity of acrylate segments. The polyacrylate polyols indeed have a bulkier, more irregular structure that reduces the regularity of hydrogen bonding and affects vibrational coupling. For present samples, this region seems to be mostly affected by the type of polyol and not particularly by the choice of HDI or PDI trimer isocyanates.

The double bands at 2937–2850 cm^−1^ can be assigned to symmetric and asymmetric CH stretching vibrations originating from the –CH_2_ groups present in aliphatic chains, or from the –CH_3_ groups [63]. The detailed insets in Figure 11 show the differences in intensity of those bands between the polyurethanes with PDI or HDI trimers, with lower CH_2_ intensity for the PDI as related to the shorter length of the aliphatic chains with 5 carbon atoms (PDI) as compared to the 6 carbon atoms (HDI). A linear trend with decreasing CH_2_ intensity as a function of PDI concentration is quantitatively confirmed by plotting the relative band intensities of -CH_2_ (2937 cm^−1^) to CN-H (1550 cm^−1^) in Supplementary Information S2 for the different samples.

The region at 1000–1200 cm^−1^ represents stretching vibrations of (O=)C–O, C–O–C and C–C skeletal vibrations in the polyol structure [64], which are sharper and better defined for the PU coatings with polyester polyol A due to the more defined structural ordering and hydrogen interactions as compared to the PUD coatings. The broadness of the peaks in PUD coatings may reflect a less ordered structure due to steric hindrance around the polyacrylate polyols. In particular, the multiple peaks at 1050 cm^−1^ and 720 cm^−1^ are characteristic of the used polyol structure: they appear in PU coatings with polyester polyol A and represent the –CH_2_ groups in the aliphatic chains of the polyol with a strongly ordered structure enhanced by hydrogen bonding. In addition, there is a slight tendency that the 720 cm^−1^ band becomes more intense as the content of HDI increases, in parallel with the length of the aliphatic chain in the trimer.

**Figure 12:**
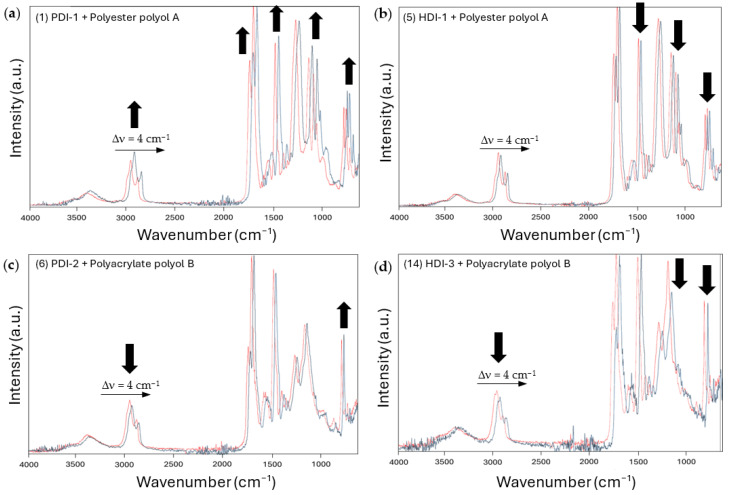
FTIR spectra of selected polyurethane coatings (red = before wear, blue = after wear; spectra shifted over Dn = 4 cm^−1^ for better visualization with black arrows indicating major intensity changes on worn samples). Sample numbers referring to Table 1: (**a**) sample 1, (**b**) sample 5, (**c**) sample 6, (**d**) sample 14.

In relation to the qualitative interpretation and variations in spectra for the native polyurethane coatings, the FTIR spectra of some polyurethane coatings after wear are illustrated in Figure 12 to identify particular chemical changes at the surface after wear.

The FTIR spectra after wear show distinct changes in relative band intensities depending on the samples with different types of isocyanates and polyols, indicating different stability and degradation mechanisms. For the PU coatings with bio-based PDI-1 and polyester polyol A (Figure 12a), the increased intensity for specific bands around 1730 cm^−1^ indicates the presence of oxidized carbonyl groups and is attributed to the creation of free oxidized species (e.g., carboxylic acids, esters). The broadening of N–H and O–H bands around 3300–3500 cm^−1^ indicates the effects of hydrolysis of the ester functions and additional hydrogen bonding. In particular, a strong increase in the C–O–C and C–H bands after wear suggests effects of the re-orientation of specific groups and enhanced exposure at the surface. These mechanisms are indeed aligned with the protective behavior and reduction in abrasive wear damage. The rearrangement of hydrogen bonds and formation of surface oxidation products are commonly observed as protective mechanisms in the wear of polyurethanes [65]. In parallel, the other studies highlighted how polyester-based PU coatings undergo clear chemical shifts in FTIR spectra, especially in the carbonyl and hydroxyl regions, due to wear and environmental stress [66]. Alternatively, the PU coatings with fossil-based HDI-1 and polyester polyol A (Figure 12b), show a reduction in specific spectral bands after wear, mainly near the C–O–C and C–H groups corresponding to the occurrence of chain scission after wear. The spectral variations after wear of polyurethane coatings with polyacrylate polyol B (Figure 12c,d) are less pronounced, though the degradation of the PUD coatings with fossil-based HDI-3 is more prevalent than for PUD coatings with bio-based PDI-2. In parallel, the other studies highlighted that polyacrylate-based systems often show less pronounced spectral changes, especially under moderate degradation conditions [59]. 

The spectral variations confirm the differences in mechanical stability and chemical changes at the surface for different coating formulations, in line with the previously discussed Tg, hardness, wear rates, and morphological surface characteristics. The chemical changes control the mechanical resistance of the polyester-polyol-based PU coatings with more beneficial surface changes for PU coatings containing bio-based PDI. Alternatively, the wear of the polyacrylate-polyol-based PUD coatings is less clearly influenced by chemical changes and more governed through mechanical damage for the PUD coatings containing PDI, while also chemical degradation is more often observed for the PUD coatings with HDI.

## 4. Conclusions

This study provides a comprehensive evaluation of hexamethylene diisocyanate (HDI) and bio-based pentamethylene diisocyanate (PDI) in formulations of polyurethane (PU) and polyurethane dispersion (PUD) coatings. The strong interdependencies between isocyanate type, polyol chemistry, and coating performance were observed by comparing curing properties, glass transition temperatures, hardness, abrasive wear rates, and chemical surface changes. The results demonstrate that bio-based PDI trimers can effectively replace HDI trimers in PU and PUD coatings without compromising mechanical integrity, and in many cases, offer superior performance.

Thermal analysis revealed that PDI-based coatings exhibit higher reactivity and glass transition temperatures in agreement with increased rigidity and crosslinking, although with slightly reduced thermal stability. Mechanical testing confirmed superior microhardness, scratch resistance, and wear resistance for PDI-based coatings, particularly when combined with polyester polyols. Surface characterization showed smoother morphologies, reduced crack density, and lower roughness on worn PU and PUD coatings with PDI in parallel with improved gloss and hydrophobicity.

The chemical integrity and crosslinking of the coatings were validated through FTIR spectroscopy, with post-wear spectra indicating beneficial surface rearrangements and oxidation in PDI-based systems. The chemical changes in worn PDI-based coatings contribute to enhanced durability and protective behavior under mechanical stress, while mechanical damage and chain scission dominate the wear degradation of HDI-based coatings.

The synergistic combination of bio-based PDI trimers with polyester polyols yields coatings with optimal thermal and mechanical properties, supporting the transition toward more sustainable and high-performance polyurethane systems. These findings contribute to the advancement of sustainable coating technologies and highlight the viability of bio-based isocyanates in industrial applications.

## Figures and Tables

**Table 1:** Overview of polyurethane coating compositions with different ratios and types of PDI or HDI isocyanates, and polyols (numbers refer to weight ratios).

Sample	Polyurethane Resin Coatings (PU)			Polyurethane Dispersion Coatings (PUD)			
	PDI-1	HDI-1	Polyester Polyol A	PDI-2	HDI-2	HDI-3	Polyacrylate Polyol B
1	1.00	-	1				
2	0.75	0.25	1				
3	0.50	0.50	1				
4	0.25	0.75	1				
5	-	1.00	1				
6				1.00	-		1
7				0.75	0.25		1
8				0.50	0.50		1
9				0.25	0.75		1
10				-	1.00		1
6′ ^(a)^				1.00		-	1
11				0.75		0.25	1
12				0.50		0.50	1
13				0.25		0.75	1
14				-		1.00	1

^(a)^ Note: samples 6 and 6′ are duplicate reference samples.

**Table 2:** Quantification of average surface roughness (Sa) and crack density on worn surfaces of polyurethane coatings.

Sample	Average Surface Roughness Sa (µm)	Crack Density (Number of Regions)	Sample	Average Surface Roughness Sa (µm)	Crack Density (Number of Regions)
1	0.283	205	6	0.30	281
2	0.337	354	7	0.34	435
3	0.358	368	8	0.43	463
4	0.378	380	9	0.56	500
5	0.419	398	10	0.79	508
			11	0.67	392
			12	0.75	402
			13	0.88	451
			14	0.93	464

## Data Availability

The original contributions presented in this study are included in the article/Supplementary Material. Further inquiries can be directed to the corresponding author.

## Supplementary Materials

The following supporting information can be downloaded at: https://www.mdpi.com/article/10.3390/polym17243301/s1, Figure S1a: FTIR spectra for polyurethane coatings with polyester polyol A. Sample numbers referring to Table 1; Figure S1b: FTIR spectra for polyurethane coatings with polyacrylate polyol B. Sample numbers referring to Table 1; Figure S1c: FTIR spectra for polyurethane coatings with polyacrylate polyol B. Sample numbers referring to Table 1; Figure S2. Qualitative analysis of FTIR spectra for polyurethane coatings with polyester polyol (◼ sample 1 to 5) and polyacrylate polyol (○ sample 6 to 11; Δ sample 6′ to 14) with different ratios of PDI relatively to HDI (for sample ratios, see Table 1).
