# Supplementary material for: Substituting Fossil-Based into Bio-Based Isocyanates for Resin and Dispersion Polyurethane Coatings: Evaluation of Thermal, Mechanical, and Chemical Performance
Source: Polymers (Basel). 2025 Dec 12;17(24):3301. doi: 10.3390/polym17243301 (PMC12737255; doi:10.3390/polym17243301)
Supplement: Supplementary file 1 [file polymers-17-03301-s001.zip › polymers-3973780-supplementary.pdf]

polymers

Supplementary Materials

Substituting Fossil-Based into Bio-based Isocyanates for Resin and Dispersion Polyurethane Coatings: Evaluation of Thermal, Mechanical, and Chemical Performance

Pieter Samyn * and Patrick Cosemans

Department of Innovations in Circular Economy and Renewable Materials, SIRRIS, Gaston Geenslaan 8, B-3001 Leuven, Belgium; patrick.cosemans@sirris.be
* Correspondence: pieter.samyn@sirris.be

Supplementary Information S1.

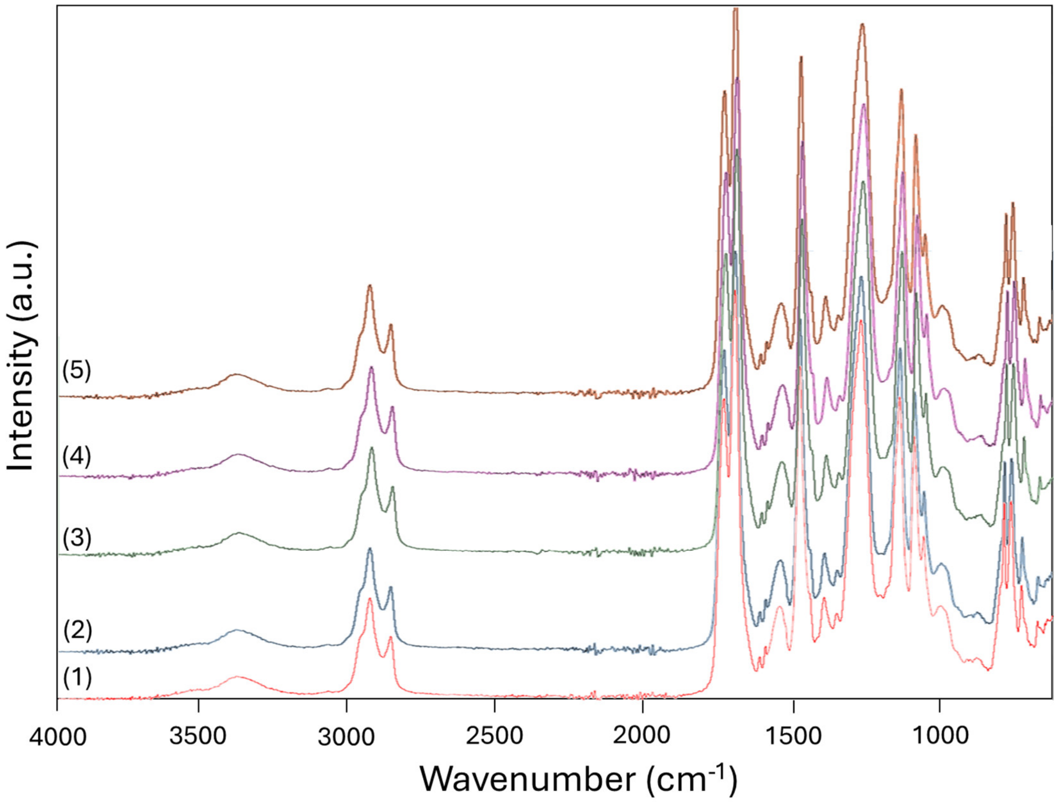

Figure S1a. FTIR spectra for polyurethane coatings with polyester polyol A. Sample numbers referring to Table 1.

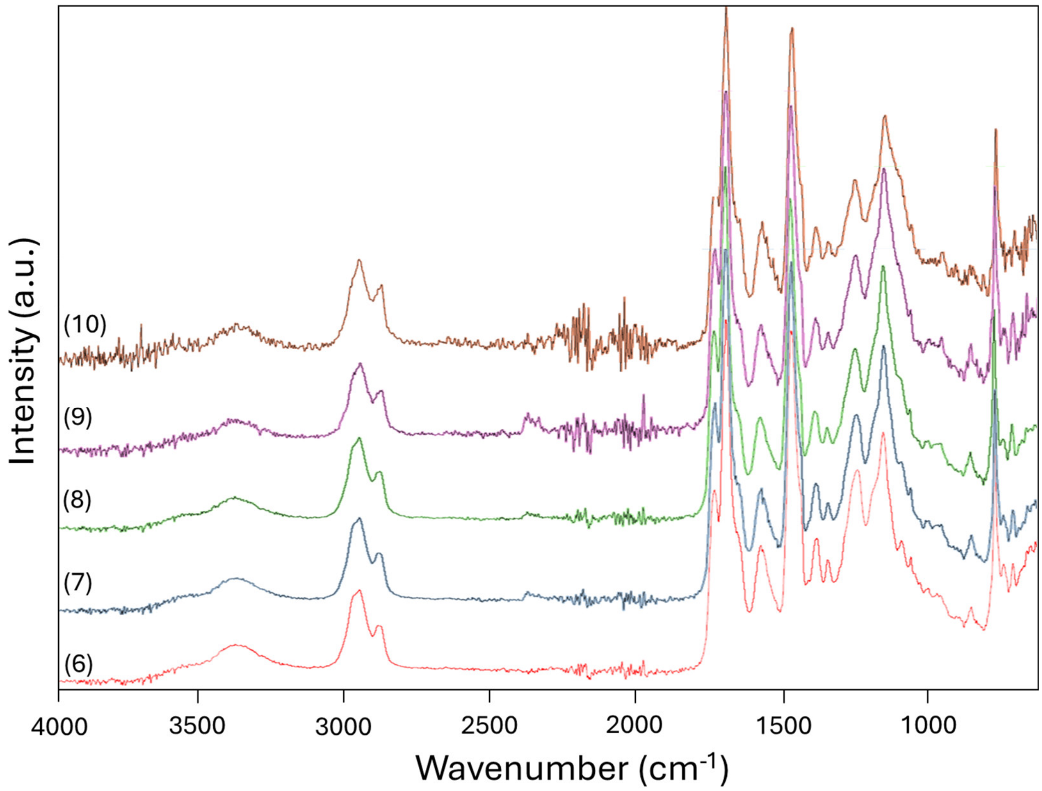

Figure S1b. FTIR spectra for polyurethane coatings with polyacrylate polyol B. Sample numbers referring to Table 1.

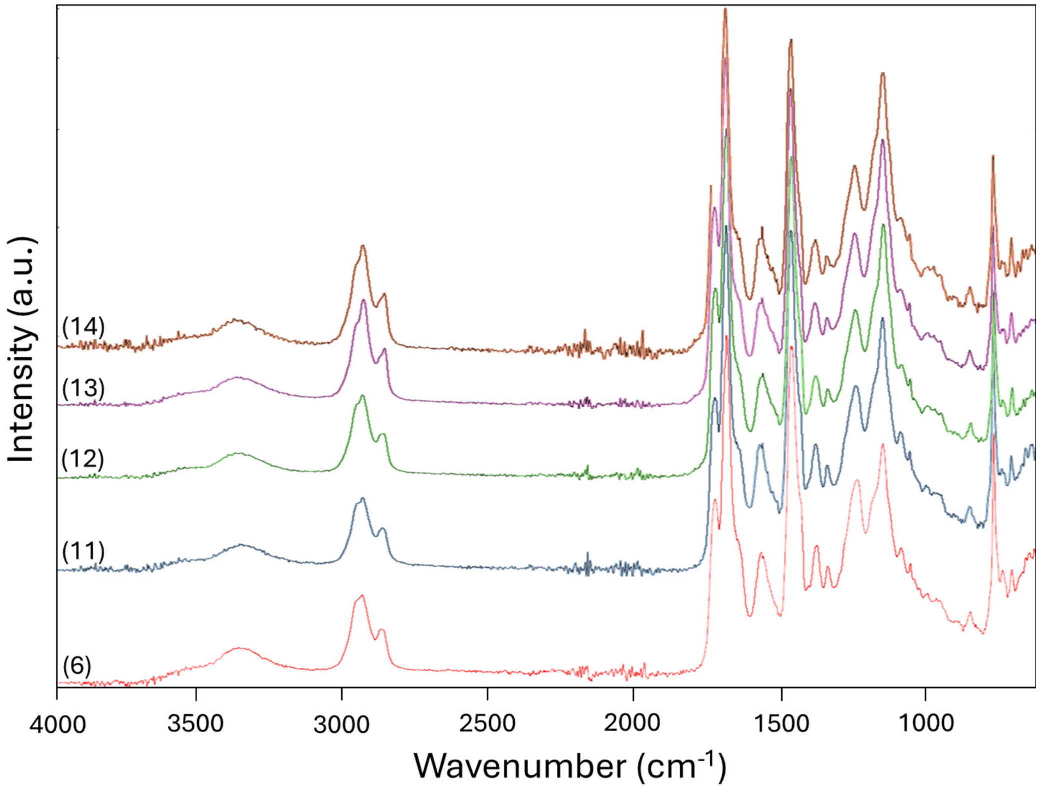

Figure S1c. FTIR spectra for polyurethane coatings with polyacrylate polyol B. Sample numbers referring to Table 1.

Supplementary Information S2.

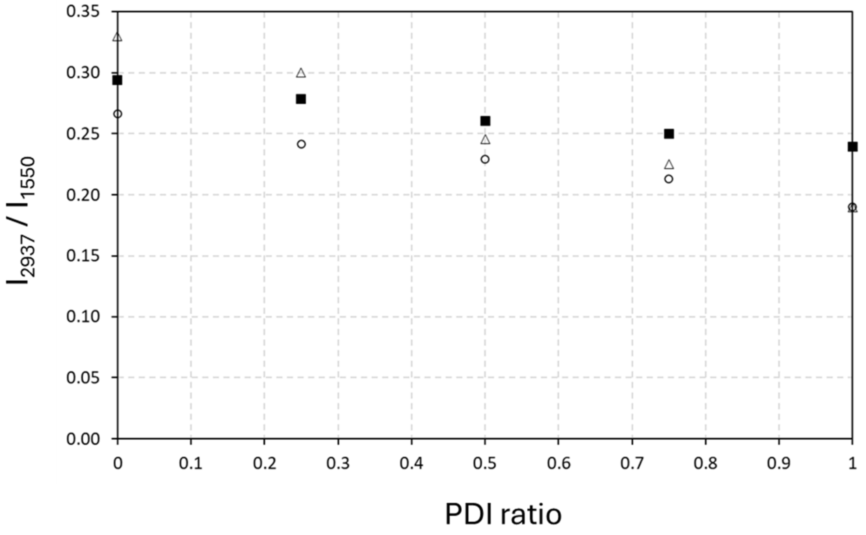

Figure S2. Qualitative analysis of FTIR spectra for polyurethane coatings with polyester polyol (■ sample 1 to 5) and polyacrylate polyol (○ sample 6 to 11; Δ sample 6′ to 14) with different ratios of PDI relatively to HDI (for sample ratios, see Table 1).
